# Avoidant/restrictive food intake disorder differs from anorexia nervosa in delay discounting

**DOI:** 10.1186/s40337-023-00958-x

**Published:** 2024-01-29

**Authors:** Casey M. Stern, Iman McPherson, Melissa J. Dreier, Kathryn Coniglio, Lilian P. Palmer, Julia Gydus, Haley Graver, Laura T. Germine, Nassim Tabri, Shirley B. Wang, Lauren Breithaupt, Kamryn T. Eddy, Jennifer J. Thomas, Franziska Plessow, Kendra R. Becker

**Affiliations:** 1 Eating Disorders Clinical and Research Program, Massachusetts General Hospital, 2 Longfellow Place, Suite 200, Boston, MA 02114 USA; 2 Neuroendocrine Unit, Massachusetts General Hospital, Boston, USA; 3 Department of Psychology, Rutgers University, New Brunswick, USA; 4 Institute for Technology in Psychiatry, McLean Hospital, Belmont, USA; 5 Department of Psychology, Carleton University, Ottawa, Canada; 6 Mental Health and Well-Being Research and Training Hub, Carleton University, Ottawa, Canada; 7 Department of Psychology, Harvard University, Cambridge, USA; 8 Department of Psychiatry, Harvard Medical School, Cambridge, USA; 9 Athinoula A. Martinos Center for Biomedical Imaging, Massachusetts General Hospital, Charlestown, USA; 10 Department of Medicine, Harvard Medical School, Cambridge, USA

**Keywords:** Anorexia nervosa, Avoidant/restrictive food intake disorder, Delay discounting, Delay of gratification, Eating disorders, Feeding and eating disorders

## Abstract

**Background:**

Avoidant/restrictive food intake disorder (ARFID) and anorexia nervosa (AN) are the two primary restrictive eating disorders; however, they are driven by differing motives for inadequate dietary intake. Despite overlap in restrictive eating behaviors and subsequent malnutrition, it remains unknown if ARFID and AN also share commonalities in their cognitive profiles, with cognitive alterations being a key identifier of AN. Discounting the present value of future outcomes with increasing delay to their expected receipt represents a core cognitive process guiding human decision-making. A hallmark cognitive characteristic of individuals with AN (vs. healthy controls [HC]) is reduced discounting of future outcomes, resulting in reduced impulsivity and higher likelihood of favoring delayed gratification. Whether individuals with ARFID display a similar reduction in delay discounting as those with AN (vs. an opposing bias towards increased delay discounting or no bias) is important in informing transdiagnostic versus disorder-specific cognitive characteristics and optimizing future intervention strategies.

**Method:**

To address this research question, 104 participants (ARFID: n = 57, AN: n = 28, HC: n = 19) completed a computerized Delay Discounting Task. Groups were compared by their delay discounting parameter (*ln*)*k*.

**Results:**

Individuals with ARFID displayed a larger delay discounting parameter than those with AN, indicating steeper delay discounting (*M* ± *SD* = −6.10 ± 2.00 vs. −7.26 ± 1.73, *p* = 0.026 [age-adjusted], Hedges’ *g* = 0.59), with no difference from HC (*p* = 0.514, Hedges’ *g* = −0.35).

**Conclusion:**

Our findings provide a first indication of distinct cognitive profiles among the two primary restrictive eating disorders. The present results, together with future research spanning additional cognitive domains and including larger and more diverse samples of individuals with ARFID (vs. AN), will contribute to identifying maintenance mechanisms that are unique to each disorder as well as contribute to the optimization and tailoring of treatment strategies across the spectrum of restrictive eating disorders.

## Abstract (plain language summary)

Avoidant/restrictive food intake disorder (ARFID) and anorexia nervosa (AN) are both restrictive eating disorders. However, the reasons for restricting food intake differ between the two diagnoses. A key question in further understanding similarities and differences between ARFID and AN is to understand whether individuals with these disorders process information and make decisions in similar or distinct ways. When humans decide between two different outcomes (e.g., a smaller immediate or a larger delayed reward), outcomes decrease in their value the farther in the future we expect to receive them (delay discounting). Individuals with AN exhibit a reduced discounting of future outcomes, which makes them more likely to forego immediate gratification for later rewards. However, whether this holds true for individuals with ARFID too (or whether they show the opposite or no bias) is unknown. Our investigation is the first to compare delay discounting between individuals with ARFID, AN, and healthy controls (HC). Our results show that individuals with ARFID show more delay discounting than those with AN, with no difference from HC. Knowing how rewards are being chosen and decisions made (and knowing differences between diagnoses) will be helpful in further optimizing and tailoring treatments for restrictive eating disorders.

## Background

Avoidant/restrictive food intake disorder (ARFID) is a psychological disorder defined by impaired growth/weight loss, nutritional deficiencies, dependence on tube feeding or nutritional supplements, and/or significant psychosocial impairment. While ARFID shares the key symptom of dietary restriction with anorexia nervosa (AN), individuals with ARFID do not describe shape and weight concerns as motives for dietary restriction. Rather, typical reasons for restriction in ARFID include sensitivity to sensory characteristics (e.g., taste, smell, texture) of food; fear of aversive consequences of eating (e.g., choking, vomiting, gastrointestinal pain); or lack of interest in eating or food.

Because cognitive profiles in AN are shown to relate to specific symptoms of the disorder, the similarities in disordered eating behaviors between ARFID and AN raise the question of whether the two conditions might also share commonalities in their cognitive profiles. Differences in cognitive functioning in AN compared to healthy controls (HC) are well-documented and considered to contribute to manifestation and maintenance of eating disorder pathology, including dietary restriction (e.g., [1, 2]). In contrast, whether/how cognitive functioning might be altered in individuals with ARFID is unknown, but important to study to inform diagnostic differences as well as possible mechanisms underlying reasons for restrictions—which may offer additional treatment targets.

Discounting the present value of future outcomes with increasing delay to their expected receipt represents a key cognitive process in guiding human decision-making, where different outcomes (e.g., rewards) with varying value and time lag to their attainment need to be weighed against each other to guide current behavior. The degree to the present value of a future outcome is discounted, and thus how future consequences of current actions are valued differs across individuals. In general, steeper discounting of the present value of a delayed outcome translates into a higher likelihood of choosing more immediate rewards over favoring long-term outcomes, and thus higher impulsivity. Conversely, less discounting results in a higher valuation of future action consequences, which has been associated with higher self-control [3, 4]. A hallmark cognitive characteristic of individuals with AN (vs. HC) is a reduced discounting of future outcomes and pronounced preference for future versus more immediate outcomes. When choosing between smaller, more immediate and larger, delayed monetary rewards, individuals with AN (compared to HC) display a stronger preference for delayed choices together with a lower delay discounting rate [3, 5, 6]. While documented independent of food, delay discounting is associated with the excessive self-control over food intake that is clinically observed in individuals with AN, in which individuals with AN often restrict consumption of highly palatable foods to support a long-term aim of excessive weight loss (e.g., [5, 7]). Moreover, in a prospective investigation following individuals with acute AN undergoing inpatient treatment to restore weight, differences in delay discounting (of monetary rewards) between individuals with AN and HC established pretreatment were no longer present following successful weight restoration [8].

Whether individuals with ARFID display a similar reduction in delay discounting as those with AN (vs. an opposing bias towards increased delay discounting or no bias) is unknown. Although food restriction in ARFID is not motivated by weight or shape concerns, individuals with ARFID similarly demonstrate difficulty eating—even when hungry—if preferred foods are not available. For example, individuals with sensory sensitivity or fear of aversive consequences as part of their ARFID presentation may resist immediate hunger and food cues for later, more desired or safer foods, and individuals with lack of interest may or may not find food rewarding at all. Thus, it is conceivable that those with ARFID may exhibit an ability to delay reward similar to what has been documented in those AN. On the other hand, clinical observation points towards people with ARFID potentially showing a preference for more short-term rewards. In line with this, a first investigation in children and adolescents with ARFID (age 6–18 years) showed lower behavioral inhibition compared to age-based norm values [9]. Unlike in AN, individuals with ARFID are not pursuing a long-term goal of controlling their shape or weight. Rather, food avoidance and restriction in ARFID seems to be characterized by inability to tolerate strong aversion or fear when it comes to food itself, with individuals instead opting for the immediate reward of avoidance. In the sensory sensitivity presentation, individuals describe avoiding tastes, textures, and smells that they experience as unpleasant or even disgusting. Individuals with a fear of aversive consequences presentation report attempts to avoid feared consequences of eating, such as choking, vomiting, or gastrointestinal pain. Finally, for those with a lack of interest presentation, it is common to experience a near or total lack of hunger cues, having a very low appetite, and feeling that eating is a chore, such that eating itself is unpleasant and preferably avoided. Across presentations, individuals with ARFID express that they wish to be able to eat a larger amount or variety of foods, but that their ARFID symptoms present an insurmountable barrier [10]. As such, it is conceivable that individuals with ARFID prefer the immediate rewarding effects of dietary avoidance/restriction compared to the delayed rewarding effects of expanding dietary intake and variety, resulting in steeper delay discounting and thus the opposing cognitive bias to the one observed in those with AN.

To the best of our knowledge, no study has examined delay discounting in individuals with ARFID, nor has any published work compared cognitive functioning in a sample with ARFID to that in a sample with AN—a comparison which is critical in order to clarify any potential relationship between dietary restriction and cognitive alterations in those with ARFID. Thus, the current study was designed to extend prior research by comparing delay discounting in a sample of individuals with ARFID, spanning both adolescents and young adults, to individuals with AN and to HC. Given that this is the first published work on delay discounting in adolescents and adults with ARFID, our primary aim was to investigate whether the ARFID group differed from the AN and HC groups. We tested this difference bi-directionally to include the possibility that individuals with ARFID could display a bias towards less pronounced discounting of future rewards (resembling the cognitive profile of AN as they resemble their restrictive eating behavior; Hypothesis #1) or a bias towards steeper discounting matching their increased impulsive behavior with increased impulsive choice (Hypothesis #2). Given that ARFID symptoms are heterogeneous across the three ARFID presentations, we additionally aimed to explore whether in the present study sample, ARFID presentation type (i.e., sensory sensitivity, fear of aversive consequences, lack of interest in eating or food) over and above the diagnosis of ARFID would be related to delay discounting rate.

## Method

### Participants

Table 1 provides demographic characteristics of the study sample.

**Table 1:** Demographic characteristics of the avoidant/restrictive food intake disorder (ARFID), anorexia nervosa (AN), and healthy control (HC) groups

		ARFID (n = 57)	AN (n = 28)	HC (n = 19)
Age, M [SD]	–	17.42 [5.38]	20.75 [3.76]	21.32 [7.60]
Sex, female N (%)	–	34 (60%)	28 (100%)	7 (37%)
Race, N (%)	American Indian/Alaska Native	1 (2%)	0 (0%)	0 (0%)
	Asian	1 (2%)	2 (7%)	2 (11%)
	Black/African American	4 (7%)	0 (0%)	3 (16%)
	Native Hawaiian/Other Pacific Islander	0 (0%)	0 (0%)	1 (5%)
	White	51 (89%)	26 (93%)	13 (68%)
Ethnicity, N (%)	Hispanic/Latinx	2 (4%)	2 (7%)	1 (5%)
	Not Hispanic/Latinx	55 (96%)	26 (93%)	18 (95%)
Body Mass Index (BMI), M [SD]	BMI percentile for age ≤ 18	36.9 [32.8]	27.6 [19.1]	58.3 [31.4]
	Absolute BMI for age > 18	24.0 [6.1]	18.5 [2.2]	28.1 [10.5]

#### ARFID and AN samples

We recruited treatment-seeking individuals with ARFID (*n* = 57) and AN (*n* = 28), ages 10–30 years from the Eating Disorders Clinical and Research Program, an outpatient eating disorders specialty clinic at Massachusetts General Hospital. To match our population of study, we purposefully included a broad age range in our sample. As an additional inclusion criterion, only individuals diagnosed with the restricting subtype of AN, as opposed to the binge-purge subtype, were considered for this investigation, given that binge eating and purging are not part of the pathology of ARFID and that we sought to compare across eating disorders where restriction is the primary symptom. Diagnoses were conferred during a routine clinical interview by each participant’s treating psychologist or psychiatrist according to *DSM-5* criteria [11, 12]. For study participants with ARFID, clinicians additionally evaluated which of the three presentations (sensory sensitivity, fear of aversive consequences, and/or lack of interest in eating or food) the participant’s symptoms were most consistent with [13]. Clinicians could select more than one presentation for each participant if appropriate, given that ARFID presentations can co-occur [14, 15]. With regard to ARFID presentation, based on clinical interviews, 45 participants endorsed sensory sensitivity, 18 endorsed fear of aversive consequences, and 24 participants endorsed lack of interest in eating or food.

#### HC

We recruited HC (*n* = 19), ages 10–30 years to match the age range of the ARFID and AN groups, who had either served as healthy controls from an NIMH-funded study (R01MH108595) investigating the neurobiology of ARFID (*n* = 4), learned about the study from Rally (a Mass General Brigham-wide recruitment platform; *n* = 4), or participated via Amazon Mechanical Turk (MTurk, an online recruitment platform for survey research; *n* = 11). HC from the neurobiology study did not meet criteria for any current psychiatric disorder on the Kiddie Schedule for Affective Disorders and Schizophrenia [16]. Adults and children from MTurk and Rally were classified as HC if they scored below clinical cut points of < 2.3 on the Eating Disorder Examination-Questionnaire [17], < 44 on the State–Trait Anxiety Inventory – Trait scale [18], < 16 on the Center for Epidemiological Studies Depression Scale [19], and < 10, 9, and 10, respectively, on the Picky Eating, Appetite, and Fear subscales of the Nine-Item ARFID Scale [13, 20]. To ensure data quality for individuals recruited through MTurk, survey settings prevented individuals from participating twice. Additionally and in line with recommendations for analyzing MTurk data [21], we ensured there were no duplicate entries by collecting MTurk worker IDs. To ensure the absence of “bots” or non-human workers in our dataset, we also embedded one or two validity checks in each measure (a practice which has been supported by Kung et al. [22]), later removing any MTurk participants who failed these validity checks [21]. Participant compensation varied by recruitment source. Clinic participants received no compensation as they completed the necessary measures as part of routine care. HC drawn from our team’s neurobiology of ARFID study received up to $300 (because the broader study involved two multi-hour visits, including not just questionnaires but also functional magnetic resonance imaging scans, test meals, and blood draws; the full protocol is outlined in [23]. MTurk and Rally participants received $1 for completing a pre-screener and $15 for completing a battery of questionnaires.

### Measures

#### Demographics

All participants self-reported their age, sex, race, and ethnicity.

#### Delay Discounting Task

Participants completed a web-based Delay Discounting Task designed for self-administration via testmybrain.org [24]. In this task, participants made repeated hypothetical decisions about whether they preferred different amounts of money now or in the future, with both the size of the monetary reward and the length of time the participant would have to wait for the reward increasing gradually. The task included seven delay periods (two weeks, one month, six months, one year, three years, five years, and ten years). Participants completed six trials for each of the seven delay periods, plus four catch trials, for a total of 46 trials. Participants who scored ≤ 50% on catch trials were ultimately excluded from analyses (n = 1 participant was excluded after implementing this criterion, which did not alter our results). Trials began with choosing between US$500 now (present outcome option) and US$1,000 (future outcome option) after the delay period in question. Based on their response, the present outcome option was adjusted in consecutive trials using the following procedure: A change amount was calculated as 500/(2^(count-1)) with count representing the trial count for the respective delay period. If a participant selected the present outcome option, the present outcome option for the consecutive trial was set as current present outcome – change amount. If a participant chose the future outcome option, the present outcome option in the following trial was calculated as present outcome + change amount. The future outcome option was kept constant at US$1,000. Four catch trials with clearly preferred reward outcomes were included to confirm participants’ attention to the task. An individual’s delay discounting parameter *k* was calculated as an average across the seven series of choices, with higher scores indicating a steeper discounting of the value of delayed rewards.

#### Eating Disorder Examination-Questionnaire (EDE-Q)

The EDE-Q [17] is a well-established measure of pathological eating behaviors and related attitudes and cognitions. Participants rate the number of days over the past 28 days in which they engaged in behaviors. The EDE-Q generates four subscale scores, and a global score is calculated by taking the average of the subscale scores. In the current study, we used a cut-off score of 2.3 on the EDE-Q Global Score [25] to rule out eating disorders other than ARFID in the ARFID and HC groups. Internal consistency for the EDE-Q in our sample was excellent (Cronbach’s alpha = 0.96).

### Data analysis

We conducted all analyses in R [26]. Packages used for analyses included: *dplyr* [27], *psych* [28], *corrplot* [29], *tidyverse* [30], *ggplot2* [31], *stringr* [32], *fmsb* [33], *rstatix* [34]. To approximate normal distribution of *k, k* values were log-transformed using natural log prior to data analysis ([*ln*]*k*). Scores for parameter (*ln*)*k* are always negative, with higher (closer to 0) scores reflecting steeper discounting of the value of delayed rewards.

To test whether the ARFID, AN, and HC groups differ in their delay discounting, we compared group means of (*ln*)*k* using an analysis of covariance (ANCOVA), with age as a covariate in all analyses. We then ran Bonferroni-corrected pairwise *t*-tests based on age-adjusted means to conduct planned comparisons with each group.

For the exploratory analysis of delay discounting within each ARFID presentation, we built a multiple linear regression model for the ARFID group regressing (*ln*)*k* on ARFID presentation type (sensory sensitivity, fear of aversive consequences, and lack of interest) and controlling for age. The three ARFID presentations were entered as dichotomous independent variables with responses coded as 0 or 1, where 1 represented having the presentation. We used multiple regression to account for overlap between presentations.

## Results

### Delay discounting in ARFID, AN, and HC groups

**Fig. 1:**
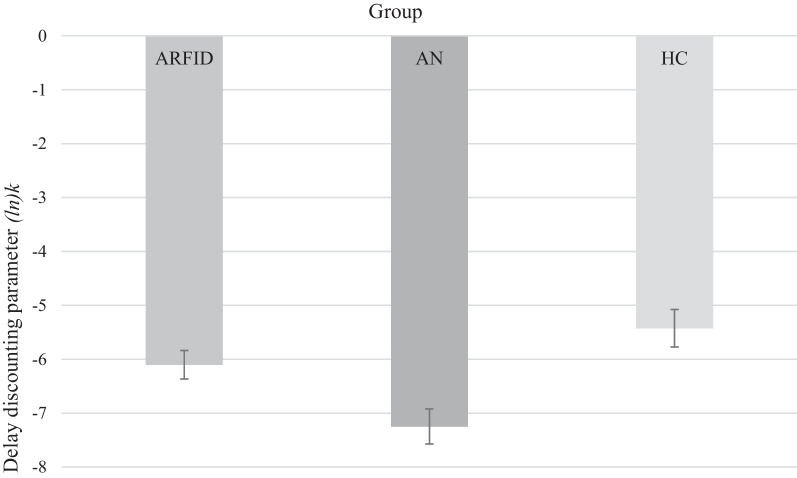
Bar graph of age-adjusted group means and between-group Analysis of Covariance (ANCOVA) with planned pairwise comparisons for the (log-transformed) delay discounting parameter (*ln*)*k*. *Note: *Error bars represent *SEM*. ARFID = avoidant/restrictive food intake disorder; AN = anorexia nervosa; HC = healthy controls

Group means and results for the hypotheses-testing ANCOVA are presented in Tables 2 and 3 and in Fig. 1. The ANCOVA showed a significant main effect of group on the delay discounting parameter (*ln*)*k* (*F*_2, 101_ = 5.93, *p* = 0.004, eta_p_^2^ = 0.11). Bonferroni-corrected post hoc pairwise comparisons revealed that the ARFID group showed a higher delay discounting parameter (*ln*)*k* than the AN group (*p* = 0.026), while no difference between the ARFID and HC groups was observed (corrected *p* = 0.51). Finally, consistent with prior literature, the AN group scored significantly higher than HC (corrected *p* = 0.004; Fig. 1). A post hoc analysis of achieved power indicated that we had sufficient statistical power to test our hypotheses (eta_p_^2^ = 0.11, 1-ß err prob = 0.88).

**Table 2:** Age-adjusted scores on the (log-transformed) delay discounting parameter (*ln*)*k* for the sample with avoidant/restrictive food intake disorder (ARFID), the sample with anorexia nervosa (AN), and the healthy control (HC) sample

	ARFID (*n* = 57)	AN (*n* = 28)	HC (*n* = 19)	*F*	Omnibus *p*
*k* score, M [SD]	0.03 [0.12]	0.006 [0.02]	0.01 [0.02]	N/A	N/A
*ln*(*k*), M [SD]	−6.1 [2]	−7.3 [1.7]	−5.4 [1.5]	5.93	0.004
Age, M [SD]	17.4 [5.4]	20.8 [3.8]	21.3 [7.6]	5.47	0.006

**Table 3:** Results of Bonferroni-corrected pairwise t-tests for the (log-transformed) delay discounting parameter (*ln*)*k* and age between the ARFID, AN, and HC groups

Parameter	ARFID–AN	AN–HC	ARFID–HC
*ln*(*k*), *p* (Hedges’ *g*)	0.026 (0.59)	0.004 (−1.09)	0.514 (−0.35)
Age, *p* (Hedges' *g*)	0.03 (−0.67)	1.00 (−0.10)	0.03 (−0.64)

### Delay discounting across ARFID presentations

Our exploratory multiple linear regression model evaluating the contribution of ARFID presentation to delay discounting was not significant (*F* = 1.99, *p* = 0.109, adjusted *R*-squared = 0.07). In other words, in our sample, none of the ARFID presentations contributed significantly to the delay discounting parameter (*ln*)*k* (B = −1.37 [*p* = 0.068] for sensory sensitivity, B = −0.64 [*p* = 0.321] for fear of aversive consequences, and B = −0.95 [*p* = 0.073] for lack of interest).

## Discussion

Alterations in preference for delayed reward are a hallmark of AN. The current research is the first to examine whether differences in delay discounting might exist in adults and children with ARFID compared to those with AN and to HC. Consistent with our Hypothesis #2, we found that those with ARFID exhibited a steeper discounting of future monetary outcomes (translating into a higher likelihood of choosing more immediate outcomes) than those with AN (with no difference to HC). This finding fits previous clinical observations. More specifically, individuals with AN may exert cognitive control to reduce food intake, thus delaying reward (i.e., food) notably compared to healthy individuals. On the other hand, those with ARFID may find food in itself less rewarding, and thus may not experience a need to utilize cognitive control resources to delay/prevent dietary intake. In other words, these findings indicate that the individual’s motivation for restriction may matter significantly in understanding disorder persistence, over and above the restriction behaviors themselves. Another possible interpretation of our findings is that preference for more immediate reward may even offer an explanation for some of the challenges many individuals with ARFID seem to face: namely, opting for the short-term reward of safe foods and avoiding negative feelings over the long-term reward of expanding dietary variety or volume. Additionally, while we did not observe a statistically significant difference in delay discounting between those with ARFID and healthy individuals, the large difference in mean scores between the two groups may be a signal to investigate other approaches—for example, examining delay discounting with severity of ARFID symptoms rather than the binary outcome of having ARFID or not. Future research following this signal (e.g., investigating delay discounting as a mediator between avoidance behaviors and disorder persistence) and attempting to further understand the possible role of delay discounting in the maintenance of ARFID is warranted.

The current research is a compelling introduction to a distinct cognitive profile of ARFID on which future research can expand. This research offers novel findings as it is the first to examine delay discounting in individuals with ARFID. We used a well-supported measure of this cognitive construct (i.e., the testmybrain.org Delay Discounting Task), which is important considering the task impurity problem common in cognitive and neuropsychological testing [1, 24]. Prior research suggests that discounting for hypothetical and real rewards produces very similar results, supporting the validity of the testmybrain.org Delay Discounting Task [35]. To date, only one published study has explored aspects of cognitive functioning in youth with ARFID [9], and to our knowledge, no prior studies have explored aspects of cognitive functioning in adults with ARFID or characterized delay discounting in any ARFID sample. The current study indicates that those with ARFID may have a cognitive profile that is distinct from those with AN. Understanding similarities and differences in the cognitive profile underlying ARFID compared to that in AN may contribute to the pursuit of improved clinical care in ARFID, given current conceptualizations of ARFID as cognitive-behavioral in nature [36]. Gaining an increased understanding of how cognition might be similar or different in those with ARFID compared to those with AN and other eating disorders treated with cognitively-oriented treatments (i.e., cognitive-behavioral therapy) may elucidate cognition treatment targets. For example, those with ARFID might exhibit some cognitive features that are similar to what is seen in AN, such as cognitive rigidity [37, 38] or all-or-nothing thinking [39]. Thinking of this nature has been observed in individuals with ARFID in an outpatient setting [10]. These and other potential cognitive similarities and differences between those with ARFID and those with AN provide a compelling avenue for future research.

Limitations of the current research should be recognized. Generalizability is a potential limitation of this research, given that our measure of delay discounting utilized monetary rewards; future research should seek to replicate these findings with food rewards, to investigate the possibility that individuals with ARFID may respond differently to rewards that are more salient to their disorder. While the overall sample was moderate in size, some presentations within the ARFID group (though only considered in a follow-up exploratory analysis) contained a relatively small number of participants (i.e., *n* = 18 for fear of aversive consequences). Further, the HC group was small due to cleaning measures applied to the data (e.g., applying an age cutoff to match the age range of the ARFID and AN groups). Replication in samples with greater numbers of both participants with ARFID and healthy control participants is needed to corroborate our findings. While all individuals in the HC group were equivalently screened for eligibility, we employed a heterogeneous sample recruited from multiple sources. Low racial and ethnic diversity bears on the generalizability of the study to diverse populations, given that these aspects of cognitive processing are known to vary between different populations and cultural contexts (e.g., [40–45]). Additionally, while we controlled for age in the present study to account for the wide developmental range of our sample (ages 10–30), developmental age is an important consideration for future research.

## Conclusions

Future research should aim to expand on this promising initial finding by investigating potential relationships between delay discounting and severity of ARFID symptoms as well as examining these constructs longitudinally. Further, there are many other domains of cognitive functioning that may be worthwhile to examine together in samples with ARFID to evaluate the full cognitive profile. Understanding similarities and differences in the cognitive mechanisms underlying ARFID compared to AN may help elucidate new maintenance mechanisms for this condition.

## Data Availability

The data that support the findings of this study are available from the corresponding author upon request.

## Abbreviations

ARFID: Avoidant/restrictive food intake disorder
AN: Anorexia nervosa
HC: Healthy control
Mturk: Amazon Mechanical Turk
EDE-Q: Eating Disorder Examination-Questionnaire
ANCOVA: Analysis of covariance
